# Supplementary material for: Impact of occupational stress on healthcare workers’ family members before and during COVID-19: A systematic review
Source: PLoS One. 2024 Sep 19;19(9):e0308089. doi: 10.1371/journal.pone.0308089 (PMC11412678; doi:10.1371/journal.pone.0308089)
Supplement: S1 File — Supporting Information file includes five supporting information documents which are described in the following: Supporting Information 1: Key Search Terms (including PsychINFO, Scopus, Medline, and Embase)Supporting Information 2: AMSTAR ChecklistSupporting Information 3: PRISMA ChecklistSupporting Information 4: CASP Results for Qualitative StudiesSupporting Information 5: AXIS Results for Quantitative Studies (DOCX) [file pone.0308089.s001.docx]

**Supporting Information**

**List of Supporting Information:**

- Supporting Information 1: Key Search Terms (including PsychINFO, Scopus, Medline, and Embase)
- Supporting Information 2: AMSTAR Checklist
- Supporting Information 3: PRISMA Checklist
- Supporting Information 4: CASP Results for Qualitative Studies
- Supporting Information 5: AXIS Results for Quantitative Studies

**Supporting Information 1:**

**PsychINFO Search**

1 exp *personnel/ (441004)

2 astronaut*.ti,ab. (557)

3 seafarer*.ti,ab. (110)

4 fisherm?n*.ti,ab. (328)

5 oil rig worker*.ti,ab. (10)

6 healthcare worker*.ti,ab. (2144)

7 healthcare staff.ti,ab. (610)

8 health professional*.ti,ab. (36117)

9 doctor*.ti,ab. (42434)

10 Nurse*.ti,ab. (74243)

11 (midwives or midwife).ti,ab. (2734)

12 paramedic*.ti,ab. (1043)

13 ambulance driver*.ti,ab. (25)

14 social worker*.ti,ab. (26185)

15 psychotherapist*.ti,ab. (15431)

16 psychologist*.ti,ab. (90608)

17 mental health professional*.ti,ab. (17644)

18 aircraft pilot*.ti,ab. (146)

19 airline pilot*.ti,ab. (314)

20 aviator*.ti,ab. (599)

21 formula one driver*.ti,ab. (1)

22 racing driver*.ti,ab. (15)

23 antarctic worker*.ti,ab. (1)

24 antarctic explorer*.ti,ab. (10)

25 antarctic expeditioner*.ti,ab. (6)

26 police officer*.ti,ab. (6218)

27 policem?n.ti,ab. (656)

28 (firefighter* or fire fighter*).ti,ab. (1573)

29 firem?n.ti,ab. (159)

30 miner*.ti,ab. (4311)

31 foreign aid worker*.ti,ab. (5)

32 missionar*.ti,ab. (1166)

33 farmer*.ti,ab. (3294)

34 journalist*.ti,ab. (3803)

35 diplomat*.ti,ab. (897)

36 high risk role*.ti,ab. (4)

37 high risk occupation*.ti,ab. (145)

38 high risk worker*.ti,ab. (15)

39 high risk profession*.ti,ab. (55)

40 deep sea diver*.ti,ab. (7)

41 construction worker*.ti,ab. (454)

42 nuclear worker*.ti,ab. (5)

43 nuclear technician*.ti,ab. (0)

44 emergency responder*.ti,ab. (201)

45 emergency personnel*.ti,ab. (130)

46 first responder*.ti,ab. (1017)

47 care home staff*.ti,ab. (112)

48 care home worker*.ti,ab. (8)

49 nursing home worker*.ti,ab. (18)

50 (search and rescue*).ti,ab. (329)

51 or/1-50 (631074)

52 Occupational Exposure/ (1299)

53 industrial accidents/ (1300)

54 dangerousness/ (1476)

55 work related illnesses/ (1180)

56 occupational stress/ (24097)

57 occupation* stress*.ti,ab. (3061)

58 hazard*.ti,ab. (25111)

59 danger*.ti,ab. (32127)

60 high risk*.ti,ab. (44651)

61 accident*.ti,ab. (24001)

62 harm*.ti,ab. (72805)

63 (injury or injuries or injured).ti,ab. (95456)

64 emergency.ti,ab. (30255)

65 high impact*.ti,ab. (1485)

66 frontline*.ti,ab. (3757)

67 or/52-66 (325767)

68 51 and 67 (56187)

69 exp family/ (329594)

70 (family or families).ti,ab. (403711)

71 spouse*.ti,ab. (20284)

72 (wives or wife).ti,ab. (14291)

73 husband*.ti,ab. (14008)

74 partner*.ti,ab. (117338)

75 child*.ti,ab. (747676)

76 parent*.ti,ab. (295612)

77 sibling*.ti,ab. (23504)

78 brother*.ti,ab. (6462)

79 sister*.ti,ab. (6463)

80 daughter*.ti,ab. (13404)

81 son*.ti,ab. (39890)

82 or/69-81 (1228989)

83 exp Mental Health/ (81969)

84 exp Well Being/ (55069)

85 emotional trauma/ or posttraumatic stress/ or trauma reactions/ or traumatic experiences/ (18074)

86 psychological stress/ (9459)

87 exp posttraumatic stress disorder/ or "stress and trauma related disorders"/ (37830)

88 acute stress disorder/ (647)

89 occupational health psychology/ (195)

90 occupational neurosis/ (30)

91 psychological stress*.ti,ab. (5891)

92 mental health*.ti,ab. (210149)

93 stress* disorder*.ti,ab. (40286)

94 (post-traumatic stress* or PTSD* or posttraumatic stress*).ti,ab. (50738)

95 compassion fatigue*.ti,ab. (1253)

96 burnout*.ti,ab. (14734)

97 vicarious trauma*.ti,ab. (999)

98 secondary trauma*.ti,ab. (1570)

99 social support*.ti,ab. (53735)

100 family support*.ti,ab. (7465)

101 coping*.ti,ab. (84481)

102 family health*.ti,ab. (2348)

103 marriage*.ti,ab. (30938)

104 interpersonal relationship*.ti,ab. (12128)

105 work schedule*.ti,ab. (1395)

106 or/83-105 (493309)

107 job satisfaction/ (21198)

108 exp Life Experiences/ (32986)

109 attitudes/ or adolescent attitudes/ or adult attitudes/ or attitude change/ or child attitudes/ or female attitudes/ or health personnel attitudes/ or occupational attitudes/ or psychologist attitudes/ or "work (attitudes toward)"/ or world view/ (112646)

110 family health*.ti,ab. (2348)

111 experience*.ti,ab. (714820)

112 support*.ti,ab. (744450)

113 impact*.ti,ab. (430590)

114 perception*.ti,ab. (326202)

115 reflection*.ti,ab. (60152)

116 opinion*.ti,ab. (52104)

117 need*.ti,ab. (656412)

118 attitude*.ti,ab. (221281)

119 belief*.ti,ab. (139786)

120 satisfaction*.ti,ab. (118497)

121 feeling*.ti,ab. (115531)

122 expectation*.ti,ab. (90337)

123 view*.ti,ab. (322994)

124 or/107-123 (2563078)

125 what happens at work comes home.m_titl. (0)

126 posttraumatic stress in children with first responders in their families.m_titl. (1)

127 (Experiences and views of frontline healthcare workers family members in the UK during the COVID-19 pandemic).m_titl. (1)

128 68 and 82 and 106 and 124 (4103)

**PsychINFO Search**

"high risk personnel*" OR "high risk staff" OR "high risk occupation*" OR "high risk role*" OR "high risk worker*" OR "high risk profession*" OR "first responder*" OR "frontline*" OR "front-line*" OR "emergency responder*" OR "emergency personnel*" OR astronaut* OR seafarer* OR police* OR "fire fighter*" OR firefighter* OR "health care worker*" OR "healthcare worker*"

OR astronaut* OR seafarer* OR fisherman OR fishermen OR "oil rig worker*" OR "healthcare staff*" OR "healthcare worker*" OR "health professional*" or doctor* OR Nurse* OR midwives OR midwife OR paramedic* OR "ambulance driver*" OR "social worker*" OR psychotherapist* OR psychologist* OR "mental health professional*" OR "aircraft pilot*" OR "airline pilot*" OR aviator* OR "formula one driver*" OR "racing driver*" OR "antarctic worker*" OR "antarctic explorer*" OR "antarctic expeditioner*" OR "police officer*" OR policeman OR policemen OR miner* OR foreign aid worker* OR missionar* OR farmer* OR journalist* OR diplomat* OR "construction worker*" OR "nuclear worker*" OR "nuclear technician*"

AND

family OR families OR spouse* OR wives OR wife OR husband* OR partner* OR child* OR parent* OR sibling* OR brother* OR sister* OR daughter* OR son*

AND

"Mental Health" OR "Well Being" OR wellbeing OR trauma* OR "psychological stress" OR "stress* disorder*" OR "post-traumatic stress*" OR PTSD* OR "posttraumatic stress*" OR "compassion fatigue*" OR burnout* OR "vicarious trauma*" OR "social support*" OR "family support*" OR coping* OR "family health*" OR marriage* OR "interpersonal relationship*" OR "work schedule*"

**Scopus Search**

"high risk personnel*" OR "high risk staff" OR "high risk occupation*" OR "high risk role*" OR "high risk worker*" OR "high risk profession*" OR "first responder*" OR "frontline*" OR "front-line*" OR "emergency responder*" OR "emergency personnel*" OR astronaut* OR seafarer* OR police* OR "fire fighter*" OR firefighter* OR "health care worker*" OR "healthcare worker*"

AND

family OR families OR spouse* OR wives OR wife OR husband* OR partner* OR child* OR parent* OR sibling* OR brother* OR sister* OR daughter* OR son*

AND

"Mental Health" OR "Well Being" OR wellbeing OR "emotional trauma*" OR "psychological trauma*" OR "psychological stress" OR "stress* disorder*" OR "post-traumatic stress*" OR PTSD* OR "posttraumatic stress*" OR "compassion fatigue*" OR burnout* OR "vicarious trauma*" OR "social support*" OR "family support*" OR coping* OR "family health*" OR marriage* OR "interpersonal relationship*" OR "work schedule*"

AND

"job satisfaction*" OR experience* OR attitude* OR view* OR support* OR impact* OR perception* OR reflection* OR opinion* OR need* OR belief* OR feeling* OR expectation*

**Medline Search**

1 exp occupational groups/

2 astronaut*.ti,ab.

3 seafarer*.ti,ab.

4 fisherm?n*.ti,ab.

5 oil rig worker*.ti,ab.

6 healthcare worker*.ti,ab.

7 healthcare staff.ti,ab.

8 health professional*.ti,ab.

9 doctor*.ti,ab.

10 Nurse*.ti,ab.

11 (midwives or midwife).ti,ab.

12 paramedic*.ti,ab.

13 ambulance driver*.ti,ab.

14 social worker*.ti,ab.

15 psychotherapist*.ti,ab.

16 psychologist*.ti,ab.

17 mental health professional*.ti,ab.

18 aircraft pilot*.ti,ab.

19 airline pilot*.ti,ab.

20 aviator*.ti,ab.

21 formula one driver*.ti,ab.

22 racing driver*.ti,ab.

23 antarctic worker*.ti,ab.

24 antarctic explorer*.ti,ab.

25 antarctic expeditioner*.ti,ab.

26 police officer*.ti,ab.

27 policem?n.ti,ab.

28 firefighter*.ti,ab.

29 firem?n.ti,ab.

30 miner*.ti,ab.

31 foreign aid worker*.ti,ab.

32 missionar*.ti,ab.

33 farmer*.ti,ab.

34 journalist*.ti,ab.

35 diplomat*.ti,ab.

36 high risk role*.ti,ab.

37 high risk occupation*.ti,ab.

38 high risk worker*.ti,ab.

39 high risk profession*.ti,ab.

40 deep sea diver*.ti,ab.

41 construction worker*.ti,ab.

42 nuclear worker*.ti,ab.

43 nuclear technician*.ti,ab.

44 emergency responder*.ti,ab.

45 first responder*.ti,ab.

46 care home staff*.ti,ab.

47 care home worker*.ti,ab.

48 nursing home worker*.ti,ab.

49 (search and rescue*).ti,ab.

50 or/1-49

51 Occupational Exposure/

52 Accidents, Occupational/

53 Occupational Injuries/

54 exp Occupational Diseases/

55 hazard*.ti,ab.

56 danger*.ti,ab.

57 high risk.ti,ab.

58 accident*.ti,ab.

59 harm*.ti,ab.

60 injury or injuries or injured).ti,ab.

61 emergency.ti,ab.

62 high impact*.ti,ab.

63 or/51-62

64 50 and 63

65 exp Family/

66 (family or families).ti,ab.

67 spouse*.ti,ab.

68 (wives or wife).ti,ab.

69 husband*.ti,ab.

70 partner*.ti,ab.

71 child*.ti,ab.

72 parent*.ti,ab.

73 sibling*.ti,ab.

74 brother*.ti,ab.

75 sister*.ti,ab.

76 daughter*.ti,ab.

77 son*.ti,ab.

78 or/65-77

79 Mental Health/

80 exp "Trauma and Stressor Related Disorders"/

81 psychological stress*.ti,ab.

82 mental health*.ti,ab.

83 stress disorder*.ti,ab.

84 (post-traumatic stress* or PTSD* or posttraumatic stress*).ti,ab.

85 compassion fatigue*.ti,ab.

86 burnout*.ti,ab.

87 vicarious trauma*.ti,ab.

88 secondary trauma*.ti,ab.

89 social support*.ti,ab.

90 family support*.ti,ab.

91 coping*.ti,ab.

92 family health*.ti,ab.

93 marriage*.ti,ab.

94 interpersonal relationship*.ti,ab.

95 work schedule*.ti,ab.

96 or/79-95

97 64 and 78 and 96

98 (Experiences and perceptions of family members of emergency first responders with post-traumatic stress disorder).m_titl.

99 Attitude/

100 Family Health/

101 experience*.ti,ab.

102 support*.ti,ab.

103 impact*.ti,ab.

104 perception*.ti,ab.

105 reflection*.ti,ab.

106 opinion*.ti,ab.

107 need*.ti,ab.

108 attitude*.ti,ab.

109 belief*.ti,ab.

110 satisfaction*.ti,ab.

111 feeling*.ti,ab.

112 expectation*.ti,ab.

113 or/99-112

114 97 and 113

**Embase Search**

120 exp *named groups by occupation/

121 astronaut*.ti,ab.

122 seafarer*.ti,ab.

123 fisherm?n*.ti,ab.

124 oil rig worker*.ti,ab.

125 healthcare worker*.ti,ab.

126 healthcare staff.ti,ab.

127 health professional*.ti,ab.

128 doctor*.ti,ab.

129 Nurse*.ti,ab.

130 (midwives or midwife).ti,ab.

131 paramedic*.ti,ab.

132 ambulance driver*.ti,ab.

133 social worker*.ti,ab.

134 psychotherapist*.ti,ab.

135 psychologist*.ti,ab.

136 mental health professional*.ti,ab.

137 aircraft pilot*.ti,ab.

138 airline pilot*.ti,ab.

139 aviator*.ti,ab.

140 formula one driver*.ti,ab.

141 racing driver*.ti,ab.

142 antarctic worker*.ti,ab.

143 antarctic explorer*.ti,ab.

144 antarctic expeditioner*.ti,ab.

145 police officer*.ti,ab.

146 policem?n.ti,ab.

147 (firefighter* or fire fighter*).ti,ab.

148 firem?n.ti,ab.

149 miner*.ti,ab.

150 foreign aid worker*.ti,ab.

151 missionar*.ti,ab.

152 farmer*.ti,ab.

153 journalist*.ti,ab.

154 diplomat*.ti,ab.

155 high risk role*.ti,ab.

156 high risk occupation*.ti,ab.

157 high risk worker*.ti,ab.

158 high risk profession*.ti,ab.

159 deep sea diver*.ti,ab.

160 construction worker*.ti,ab.

161 nuclear worker*.ti,ab.

162 nuclear technician*.ti,ab.

163 emergency responder*.ti,ab.

164 first responder*.ti,ab.

165 care home staff*.ti,ab.

166 care home worker*.ti,ab.

167 nursing home worker*.ti,ab.

168 (search and rescue*).ti,ab.

169 or/120-168

170 occupational hazard/

171 occupational exposure/

172 occupational accident/

173 exp *occupational disease/

174 occupational stress*.ti,ab.

175 hazard*.ti,ab.

176 danger*.ti,ab.

177 high risk*.ti,ab.

178 accident*.ti,ab.

179 harm*.ti,ab.

180 (injury or injuries or injured).ti,ab.

181 emergency.ti,ab.

182 high impact*.ti,ab.

183 frontline*.ti,ab.

184 or/170-183

185 169 and 184

186 exp *family/

187 (family or families).ti,ab.

188 spouse*.ti,ab.

189 (wives or wife).ti,ab.

190 husband*.ti,ab.

191 partner*.ti,ab.

192 child*.ti,ab.

193 parent*.ti,ab.

194 sibling*.ti,ab.

195 brother*.ti,ab.

196 sister*.ti,ab.

197 daughter*.ti,ab.

198 son*.ti,ab.

199 or/186-198

200 mental health/ or psychological well-being/

201 psychotrauma/

202 exp *mental stress/

203 posttraumatic stress disorder/

204 psychological stress*.ti,ab.

205 mental health*.ti,ab.

206 stress* disorder*.ti,ab.

207 (post-traumatic stress* or PTSD* or posttraumatic stress*).ti,ab.

208 compassion fatigue*.ti,ab.

209 burnout*.ti,ab.

210 vicarious trauma*.ti,ab.

211 secondary trauma*.ti,ab.

212 social support*.ti,ab.

213 family support*.ti,ab.

214 coping*.ti,ab.

215 family health*.ti,ab.

216 marriage*.ti,ab.

217 interpersonal relationship*.ti,ab.

218 work schedule*.ti,ab.

219 or/200-218

220 experience/

221 job experience/

222 work experience/

223 personal experience/

224 exp *attitude/

225 family health/

226 experience*.ti,ab.

227 support*.ti,ab.

228 impact*.ti,ab.

229 perception*.ti,ab.

230 reflection*.ti,ab.

231 opinion*.ti,ab.

232 need*.ti,ab.

233 attitude*.ti,ab.

234 belief*.ti,ab.

235 satisfaction*.ti,ab.

236 feeling*.ti,ab.

237 expectation*.ti,ab.

238 view*.ti,ab.

239 or/220-238

240 185 and 199 and 219 and 239

**Supporting Information 2:**

AMSTAR Checklist

| \| **Impact of Occupational Stress on Families of HCWs is a High quality review** \| \| \| --- \| --- \| \| **1. Did the research questions and inclusion criteria for the review include the components of PICO?** \| Yes Yes Yes Yes Yes \| \|  \| \| |
| --- | --- | --- | --- | --- | --- | --- |
| \| **2. Did the report of the review contain an explicit statement that the review methods were established prior to the conduct of the review and did the report justify any significant deviations from the protocol?** \| YesPartial YesYesYesYesYesYesYes \| \| --- \| --- \| |
|  |
| \| **3. Did the review authors explain their selection of the study designs for inclusion in the review?** \| Yes  Yes \| \| --- \| --- \| |
|  |
| \| **4. Did the review authors use a comprehensive literature search strategy?** \| Yes Partial Yes Yes Yes Yes Yes Yes Yes Yes Yes \| \| --- \| --- \| |
|  |
| \| **5. Did the review authors perform study selection in duplicate?** \| Yes Yes \| \| --- \| --- \| |
|  |
| \| **6. Did the review authors perform data extraction in duplicate?** \| Yes Yes \| \| --- \| --- \| |
|  |
| \| **7. Did the review authors provide a list of excluded studies and justify the exclusions?** \| Yes Partial Yes Yes Yes \| \| --- \| --- \| |
|  |
| \| **8. Did the review authors describe the included studies in adequate detail?** \| Partial Yes Yes Yes Yes Yes Yes Yes Yes Yes Yes \| \| --- \| --- \| |
|  |
| \| **9. Did the review authors use a satisfactory technique for assessing the risk of bias (RoB) in individual studies that were included in the review?** \|  \| \| --- \| --- \| \| **RCT** \| 0 \| \|  \|  \| \| **NRSI** \| Partial Yes       Yes  Yes \| |
|  |
| \| **10. Did the review authors report on the sources of funding for the studies included in the review?** \| Yes Yes \| \| --- \| --- \| |
|  |
| \| **11. If meta-analysis was performed did the review authors use appropriate methods for statistical combination of results?** \|  \| \| --- \| --- \| \| **RCT** \| 0 \| \|  \|  \| \| **NRSI** \| 0 \| |
|  |
| \| **12. If meta-analysis was performed, did the review authors assess the potential impact of RoB in individual studies on the results of the meta-analysis or other evidence synthesis?** \| 0 \| \| --- \| --- \| |
|  |
| \| **13. Did the review authors account for RoB in individual studies when interpreting/ discussing the results of the review?** \| Yes  Yes \| \| --- \| --- \| |
|  |
| \| **14. Did the review authors provide a satisfactory explanation for, and discussion of, any heterogeneity observed in the results of the review?** \| Yes  Yes \| \| --- \| --- \| |
|  |
| \| **15. If they performed quantitative synthesis did the review authors carry out an adequate investigation of publication bias (small study bias) and discuss its likely impact on the results of the review?** \| 0 \| \| --- \| --- \| |
|  |
| \| **16. Did the review authors report any potential sources of conflict of interest, including any funding they received for conducting the review?** \| Yes Yes \| \| --- \| --- \| |
|  |
| To cite this tool: Shea BJ, Reeves BC, Wells G, Thuku M, Hamel C, Moran J, Moher D, Tugwell P, Welch V, Kristjansson E, Henry DA. AMSTAR 2: a critical appraisal tool for systematic reviews that include randomised or non-randomised studies of healthcare interventions, or both. BMJ. 2017 Sep 21;358:j4008. |

**Supporting Information 3:**

**PRISMA Checklist**

| **Section and Topic** | **Item #** | **Checklist item** | **Location where item is reported** |
| --- | --- | --- | --- |
| **TITLE** | | |  |
| Title | 1 | Identify the report as a systematic review. | Page 1 |
| **ABSTRACT** | | |  |
| Abstract | 2 | See the PRISMA 2020 for Abstracts checklist. | Page 2 |
| **INTRODUCTION** | | |  |
| Rationale | 3 | Describe the rationale for the review in the context of existing knowledge. | Page 4 |
| Objectives | 4 | Provide an explicit statement of the objective(s) or question(s) the review addresses. | Page 4 |
| **METHODS** | | |  |
| Eligibility criteria | 5 | Specify the inclusion and exclusion criteria for the review and how studies were grouped for the syntheses. | Page 6 |
| Information sources | 6 | Specify all databases, registers, websites, organisations, reference lists and other sources searched or consulted to identify studies. Specify the date when each source was last searched or consulted. | Page 5 |
| Search strategy | 7 | Present the full search strategies for all databases, registers and websites, including any filters and limits used. | Page 5-6 |
| Selection process | 8 | Specify the methods used to decide whether a study met the inclusion criteria of the review, including how many reviewers screened each record and each report retrieved, whether they worked independently, and if applicable, details of automation tools used in the process. | Page 6-7 |
| Data collection process | 9 | Specify the methods used to collect data from reports, including how many reviewers collected data from each report, whether they worked independently, any processes for obtaining or confirming data from study investigators, and if applicable, details of automation tools used in the process. | Page 7-8 |
| Data items | 10a | List and define all outcomes for which data were sought. Specify whether all results that were compatible with each outcome domain in each study were sought (e.g. for all measures, time points, analyses), and if not, the methods used to decide which results to collect. | Page 8 |
|  | 10b | List and define all other variables for which data were sought (e.g. participant and intervention characteristics, funding sources). Describe any assumptions made about any missing or unclear information. | Page 8-9 |
| Study risk of bias assessment | 11 | Specify the methods used to assess risk of bias in the included studies, including details of the tool(s) used, how many reviewers assessed each study and whether they worked independently, and if applicable, details of automation tools used in the process. | Page 22-23-24 |
| Effect measures | 12 | Specify for each outcome the effect measure(s) (e.g. risk ratio, mean difference) used in the synthesis or presentation of results. | - |
| Synthesis methods | 13a | Describe the processes used to decide which studies were eligible for each synthesis (e.g. tabulating the study intervention characteristics and comparing against the planned groups for each synthesis (item #5)). | Page 7-8 |
|  | 13b | Describe any methods required to prepare the data for presentation or synthesis, such as handling of missing summary statistics, or data conversions. | Page 7-8 |
|  | 13c | Describe any methods used to tabulate or visually display results of individual studies and syntheses. | Page 7-8 and Page 24 |
|  | 13d | Describe any methods used to synthesize results and provide a rationale for the choice(s). If meta-analysis was performed, describe the model(s), method(s) to identify the presence and extent of statistical heterogeneity, and software package(s) used. | - |
|  | 13e | Describe any methods used to explore possible causes of heterogeneity among study results (e.g. subgroup analysis, meta-regression). | - |
|  | 13f | Describe any sensitivity analyses conducted to assess robustness of the synthesized results. | - |
| Reporting bias assessment | 14 | Describe any methods used to assess risk of bias due to missing results in a synthesis (arising from reporting biases). | Page 6-7-8 |
| Certainty assessment | 15 | Describe any methods used to assess certainty (or confidence) in the body of evidence for an outcome. | - |
| **RESULTS** | | |  |
| Study selection | 16a | Describe the results of the search and selection process, from the number of records identified in the search to the number of studies included in the review, ideally using a flow diagram. | Page 8-9 |
|  | 16b | Cite studies that might appear to meet the inclusion criteria, but which were excluded, and explain why they were excluded. | Page 9 |
| Study characteristics | 17 | Cite each included study and present its characteristics. | Page from 9 to 21 |
| Risk of bias in studies | 18 | Present assessments of risk of bias for each included study. | Page 22-23-24 |
| Results of individual studies | 19 | For all outcomes, present, for each study: (a) summary statistics for each group (where appropriate) and (b) an effect estimate and its precision (e.g. confidence/credible interval), ideally using structured tables or plots. | -From page 11 to page 21  -From Page 25 to 33 |
| Results of syntheses | 20a | For each synthesis, briefly summarise the characteristics and risk of bias among contributing studies. | -From page 11 to page 21 |
|  | 20b | Present results of all statistical syntheses conducted. If meta-analysis was done, present for each the summary estimate and its precision (e.g. confidence/credible interval) and measures of statistical heterogeneity. If comparing groups, describe the direction of the effect. | From page 34 to 44 |
|  | 20c | Present results of all investigations of possible causes of heterogeneity among study results. | From page 34 to 44 |
|  | 20d | Present results of all sensitivity analyses conducted to assess the robustness of the synthesized results. | - |
| Reporting biases | 21 | Present assessments of risk of bias due to missing results (arising from reporting biases) for each synthesis assessed. | From page 34 to 44 |
| Certainty of evidence | 22 | Present assessments of certainty (or confidence) in the body of evidence for each outcome assessed. | - |
| **DISCUSSION** | | |  |
| Discussion | 23a | Provide a general interpretation of the results in the context of other evidence. | Page 44-45 |
|  | 23b | Discuss any limitations of the evidence included in the review. | Page 48 |
|  | 23c | Discuss any limitations of the review processes used. | Page 48-49 |
|  | 23d | Discuss implications of the results for practice, policy, and future research. | Page 49 |
| **OTHER INFORMATION** | | |  |
| Registration and protocol | 24a | Provide registration information for the review, including register name and registration number, or state that the review was not registered. | Page 1 and Page 5 |
|  | 24b | Indicate where the review protocol can be accessed, or state that a protocol was not prepared. | Page 1 and page 5 |
|  | 24c | Describe and explain any amendments to information provided at registration or in the protocol. | Page 50 |
| Support | 25 | Describe sources of financial or non-financial support for the review, and the role of the funders or sponsors in the review. | Page 50 |
| Competing interests | 26 | Declare any competing interests of review authors. | Page 50 |
| Availability of data, code and other materials | 27 | Report which of the following are publicly available and where they can be found: template data collection forms; data extracted from included studies; data used for all analyses; analytic code; any other materials used in the review. | Page 50 |

**Supporting Information 4:**

**CASP results for each qualitative study**

| **Author and Year** | **1. Was there a clear statement of the aims of the research?** | **2. Is a qualitative methodology appropriate?** | **3. Was the research design appropriate to address the aims of the research?** | **4. Was the recruitment strategy appropriate to the aims of the research?** | **5. Were the data collected in a way that addressed the research issue?** | **6. Has the relationship between researcher and participants been adequately considered?** | **7. Have ethical issues been taken into consideration?** | **8. Was the data analysis sufficiently rigorous?** | **9. Is there a clear statement of findings?** | **10. How valuable is the research?** |
| --- | --- | --- | --- | --- | --- | --- | --- | --- | --- | --- |
| Banitalebi et al., (2021) | Yes | Yes | No | Yes | Yes | Yes | No | Yes | Yes | Yes |
| Banitalebi et al., (2022) | Yes | Yes | No | Yes | Yes | No | No | Yes | Yes | Yes |
| Chandler-Jeanville et al., (2021) | Yes | Yes | Yes | Yes | Yes | Yes | No | Yes | Yes | Yes |
| Chua et al., (2021) | Yes | Yes | No | Yes | No | Yes | No | Yes | Yes | Yes |
| Emmett et al., (2013) | Yes | Yes | Yes | Yes | Yes | Yes | Yes | Yes | Yes | Yes |
| Ericson-Lidman et al., (2010) | Yes | Yes | Yes | Yes | Yes | Yes | Yes | Yes | Yes | Yes |
| Feng et al., (2020) | Yes | Yes | No | Yes | yes | No | No | Yes | Yes | Yes |
| Goud et al., (2021) | Yes | Yes | No | Yes | No | No | No | Yes | Yes | Yes |
| Henry et al., (2023) | Yes | Yes | No | Yes | Yes | y | Yes | Yes | Yes | Yes |
| King et al., (2014) | Yes | Yes | No | Yes | Yes | Yes | No | Yes | Yes | Yes |
| Lorenzo et al., (2020) | Yes | Yes | No | Yes | Yes | Yes | No | Yes | Yes | No |
| Mohammadi et al., (2022) | Yes | Yes | Yes | Yes | Yes | Yes | Yes | Yes | Yes | Yes |
| Regehr, C. D. (2005) | Yes | Yes | Yes | Yes | Yes | Yes | Yes | Yes | Yes | Yes |
| Roth et al., 2009 | Yes | Yes | Yes | Yes | Yes | Yes | Yes | Yes | Yes | Yes |
| Sachdeva et al., (2022) | Yes | Yes | No | Yes | Yes | Yes | Yes | Don't know | Yes | Yes |
| Schaffer et al., (2022) | Yes | Yes | Yes | Yes | Yes | No | Yes | Can't tell | Yes | Yes |
| Sheen et al., (2022) | Yes | Yes | Yes | Yes | Yes | Yes | Yes | Yes | Yes | Yes |
| Tekin et al., (2022) | Yes | Yes | Yes | Yes | Yes | Yes | Yes | Yes | Yes | Yes |
| Tüğen et al., (2023) | Yes | Yes | Yes | Yes | Yes | Yes | Yes | Yes | Yes | Yes |
| Ying et al., (2020) | Yes | Yes | Yes | Yes | Yes | Yes | Yes | Yes | Yes | Yes |

**Supporting Information 5:**

**AXIS results for quantitative studies**

| **Author and Year** | **Were the aims/objectives of the study clear?** | **Was the study design appropriate for the stated aim(s)?** | **Was the sample size justified?** | **Was the target/reference population clearly defined? (Is it clear who the research was about?)** | **Was the sample frame taken from an appropriate population base so that it closely represented the target/reference population under investigation?** | **Was the selection process likely to select subjects/participants that were representative of the target/reference population under investigation?** | **Were measures undertaken to address and categorise non-responders?** | **Were the risk factor and outcome variables measured appropriate to the aims of the study?** | **Were the risk factor and outcome variables measured correctly using instruments/measurements that had been trialled, piloted or published previously?** | **Is it clear what was used to determined statistical significance and/or precision estimates? (e.g. p-values, confidence intervals)** | **Were the methods (including statistical methods) sufficiently described to enable them to be repeated?** | **Were the basic data adequately described?** | **Does the response rate raise concerns about non-response bias?** | **If appropriate, was information about non-responders described?** | **Were the results internally consistent?** | **Were the results presented for all the analyses described in the methods?** | **Were the authors' discussions and conclusions justified by the results?** | **Were the limitations of the study discussed?** | **Were there any funding sources or conflicts of interest that may affect the authors’ interpretation of the results?** | **Was ethical approval or consent of participants obtained?** |
| --- | --- | --- | --- | --- | --- | --- | --- | --- | --- | --- | --- | --- | --- | --- | --- | --- | --- | --- | --- | --- |
| Alexander et al., (1996) | Yes (Y) | Y | No (N) | Y | Y | Y | Don’t Know (DK) | Y | Y | Y | Y | Y | N | N | Y | Y | Y | Y | DK | DK |
| Alrutz, et al., (2020) | Y | Y | N | Y | N | N | DK | Y | Y | N | N | Y | Y | Y | DK | Y | Y | Y | N | Y |
| Ames et al., (2013) | Y | Y | DK | Y | Y | Y | DK | Y | Y | Y | Y | Y | N | Y | Y | Y | Y | Y | Y | Y |
| Banitalebi et al., (2021) | Y | Y | N | Y | Y | Y | N | Y | Y | Y | Y | Y | Y | N | Y | Y | Y | N | N | Y |
| Banitalebi et al., (2022) | Y | Y | N | Y | Y | N | N | Y | Y | Y | Y | Y | Y | N | Y | Y | Y | Y | N | Y |
| Beard et al., (2013) | Y | Y | N | Y | Y | DK | Y | Y | Y | Y | Y | Y | N | N | Y | Y | Y | Y | N | Y |
| Beehr et al., (1995) | Y | Y | N | Y | Y | Y | Y | Y | Y | Y | Y | Y | Y | Y | Y | Y | Y | Y | N | Y |
| Brimhall et al., (2019) | Y | Y | N | Y | Y | N | Y | Y | Y | Y | Y | Y | Y | N | Y | Y | Y | Y | N | Y |
| Chua et al., (2021) | Y | Y | N | Y | N | Y | N | Y | Y | Y | Y | Y | Y | N | Y | Y | Y | Y | N | Y |
| Costa et al., (2019) | Y | Y | Y | Y | Y | DK | N | Y | Y | Y | Y | Y | Y | N | Y | Y | Y | Y | N | DK |
| Cunradi et al., (2009) | Y | Y | N | Y | Y | Y | Y | Y | Y | Y | Y | Y | Y | Y | Y | Y | Y | Y | N | Y |
| Cunradi et al., (2009) | Y | Y | N | Y | Y | Y | Y | Y | Y | Y | Y | Y | Y | Y | Y | Y | Y | Y | N | Y |
| Davidson et al., (2006) | Y | Y | N | Y | Y | Y | Y | Y | Y | Y | Y | Y | Y | Y | Y | Y | Y | Y | N | Y |
| Duarte et al (2006) | Y | Y | N | Y | Y | Y | N | Y | Y | Y | Y | Y | Y | N | Y | Y | Y | Y | N | N |
| Feng et al., (2020) | Y | Y | N | Y | Y | N | N | Y | Y | Y | Y | Y | Y | N | Y | Y | Y | Y | N | Y |
| Friese et al., (2020) | Y | Y | N | Y | Y | Y | N | Y | Y | Y | Y | Y | N | N | Y | Y | Y | Y | N | Y |
| Goud et al., (2021) | Y | Y | N | Y | N | N | N | Y | Y | Y | Y | Y | Y | N | DK | Y | Y | Y | N | Y |
| Horan et al., (2012) | Y | Y | N | Y | N | N | N | Y | Y | Y | Y | Y | Y | N | Y | Y | Y | Y | N | N |
| Hoven et al., (2009) | Y | Y | N | Y | Y | Y | DK | Y | Y | DK | N | Y | Y | N | Y | Y | DK | Y | N | Y |
| Karaffa et al., (2015) | Y | Y | Y | Y | N | Y | N | Y | DK | Y | Y | Y | Y | N | Y | Y | Y | Y | N | Y |
| Leon & Sandal (2003) | Y | Y | N | Y | Y | N | N | Y | Y | Y | Y | Y | Y | N | Y | Y | Y | Y | N | Y |
| Meffert et al., (2014) | Y | Y | N | Y | Y | N | DK | Y | Y | Y | Y | Y | DK | DK | Y | Y | Y | Y | N | Y |
| Morris et al., (1998) | Y | Y | N | Y | Y | Y | Y | Y | Y | Y | Y | Y | N | N | Y | Y | Y | Y | N | Y |
| Pfefferbaum et al., (2002) | Y | Y | N | Y | Y | N | N | Y | Y | Y | Y | Y | Y | N | Y | Y | Y | Y | N | Y |
| Pfefferbaum et al., (2006) | Y | Y | N | Y | Y | N | DK | Y | Y | Y | Y | Y | Y | DK | Y | Y | Y | Y | N | N |
| Roberts et al., (2013) | Y | Y | N | Y | Y | Y | Y | Y | Y | Y | Y | Y | Y | Y | Y | Y | Y | Y | N | Y |
| Roberts et al., (2001) | Y | Y | N | Y | Y | Y | Y | Y | Y | Y | Y | Y | DK | Y | Y | Y | Y | Y | N | Y |
| Sachdeva et al., (2022) | Y | Y | N | Y | Y | Y | Y | Y | Y | DK | Y | Y | Y | Y | DK | N | Y | Y | Y | Y |
| Slišković et al., (2019) | Y | Y | N | Y | Y | Y | DK | Y | Y | Y | Y | Y | DK | N | Y | Y | Y | Y | N | Y |
| Sprung et al., (2016) | Y | Y | N | Y | Y | Y | DK | Y | Y | Y | Y | Y | Y | N | Y | Y | Y | Y | N | Y |
| Taylor et al., (1987) | Y | Y | N | Y | Y | Y | Y | Y | Y | Y | Y | N | N | Y | Y | Y | Y | Y | N | Y |
| Uchida et al., (2018) | Y | Y | N | Y | Y | Y | Y | Y | Y | Y | Y | Y | N | N | Y | Y | Y | Y | N | Y |
| Ulven et al., (2007) | Y | Y | N | Y | Y | Y | Y | Y | Y | Y | Y | Y | Y | Y | Y | Y | Y | Y | N | Y |
| Ziello et al., (2014) | Y | Y | N | Y | Y | Y | DK | Y | Y | Y | Y | Y | DK | N | Y | Y | Y | DK | N | Y |
