# Supplementary material for: Impact of occupational stress on healthcare workers’ family members before and during COVID-19: A systematic review
Source: PLoS One. 2024 Sep 19;19(9):e0308089. doi: 10.1371/journal.pone.0308089 (PMC11412678; doi:10.1371/journal.pone.0308089)
Supplement: Attachment — Submitted filename: Comments on PONE-D-24-09879.docx [file pone.0308089.s002.docx]

**I am pleased to review manuscript ID PONE-D-24-09879. I appreciate the authors' remarkable work. However, I have provided some critical comments below to help improve the paper.**

**Best of luck,**

**Your Reviewer**

**Comments and Recommendations**

**General Comments**

- **Title and Abstract**:
  - The title is somewhat confusing, especially when referencing "before and during COVID-19." This suggests the study is solely about COVID-19 impacts post-2020, yet the search includes studies from 1980 and papers from 2005 onward. Consider adding a timeline in the title to align with the study results as per PubMed.
  - The abstract is clear and well-organized.

**Methods**

- Please add the heading of Study Design and Registration for the first paragraph of method.
- Please consider cite the PRISMA checklist guidelines in the method section.
- **Study Characteristics**:
  - Include details on the highest and lowest sample sizes in the included studies, total number of participants, mean age, and age range from the youngest to oldest participant.
- **Tables**:
  - **Table 2: Characteristics of the Included Studies**:
    - Add the first author’s last name and "et al." for studies with three or more authors.
    - Specify the specialties of HCWs (e.g., nurses, doctors, dentists) as you have only mentioned HCWs in general for some studies.
  - **Table 5: Measures Used**:
    - Standardize the use of acronyms (e.g., GAD) and provide full terms and detailed explanations in the Result section as suggested in my following comment.
  - Avoid redundancy maybe by removing the "main findings" column from Table 2 or justify its presence to prevent confusion and duplication with Table 5.

**Results and Discussion**

- **Paragraph on Measures Used**:
  - Include a detailed paragraph in the results section summarizing the various measures and diagnostic tools used across the included studies.
- **Strengths and Limitations of Included Papers**:
  - Clarify the point about the literature gap regarding research on partners and spouses in same-sex relationships, parents, and siblings of HCWs to ensure readers understand the context clearly.
