# Supplementary material for: Impact of occupational stress on healthcare workers’ family members before and during COVID-19: A systematic review
Source: PLoS One. 2024 Sep 19;19(9):e0308089. doi: 10.1371/journal.pone.0308089 (PMC11412678; doi:10.1371/journal.pone.0308089)
Supplement: Attachment — Submitted filename: .Response to Reviewers.docx [file pone.0308089.s003.docx]

Sahra Tekin

UCL Division of Psychiatry

6^th^ Floor, Maple House

149 Tottenham Court Road

London W1T 7NF

**PLOS ONE**

01 July 2024

Dear Academic Editor,

**LONDON’S GLOBAL UNIVERSITY**

**Re: Manuscript No. PONE-D-24-09879**

**Impact of Occupational Stress on Healthcare Workers' Family Members Before and During the COVID-19: A systematic review**

Thank you to you and your reviewers for your feedback on our manuscript. We are very pleased to submit a revised manuscript incorporating the reviewers’ suggestions. We have provided a point-by-point response to how we have addressed each comment in the table below.

| **Reviewers’ comments** | **Authors’ Response** | **Manuscript page number** |
| --- | --- | --- |
| ***Comments from Reviewer #1*** | | |
| Please explain why Lorenzo and Carrisi (2020) is included in this SR? Seems this paper is not talking about mental health / stress related issue. | Thank you for your comment. The reason that we included this study in our review is, our study also highlights the health risk of family members of HCWs due to contamination. Specifically, studies which were conducted during and after the COVID-19 pandemic reported that the serious health risk associated with exposure to the virus may impact the mental health and wellbeing of both the HCWs and their family members. For this reason, we think it is significant to include this article which provides quantitative evidence to show how significantly high the health risk is for the HCWs’ family members as well. We apologise for the lack of clarity. | - |
| Please provide AXIS total scores or percentage scores of each included papers to support your comment that most of the studies included in this review met the criteria for high-quality research. | Thank you very much for your comment. We have provided detailed information about the qualities of the included studies in the Supplementary materials. | See Supplementary materials |
| ***Comments from Reviewer #2*** | | |
| ***Title and Abstract:***  -The title is somewhat confusing, especially when referencing "before and during COVID-19." This suggests the study is solely about COVID-19 impacts post-2020, yet the search includes studies from 1980 and papers from 2005 onward. Consider adding a timeline in the title to align with the study results as per PubMed. | Thank you for pointing this out. Reviewer 3 also picked up on the lack of clarity in the title and suggested an alternative title. For this reason, we have revised the title based on the comments. | -1 |
| - The abstract is clear and well-organized. | Thank you for your positive feedback. | - |
| ***Methods:***  - Please add the heading of Study Design and Registration for the first paragraph of the method. | Thank you for this suggestion. We have included the following subtitle in the Method: “2.1. Study Design and Registration”. | -5 |
| - Please consider cite the PRISMA checklist guidelines in the method section. | Thank you for your comment. We have cited the PRISMA checklist guidelines in 2.1. Study Design and Registration. | -5 |
| ***Study Characteristics:***  -Include details on the highest and lowest sample sizes in the method section. | We have included two sentences to provide details about the smallest and the largest sample sizes amongst both included qualitative and quantitative studies. | -9 |
| ***Tables:***  ***Table 2: Characteristics of the Included Studies***  -Add the first author’s last name and et al., for studies with three or more authors.  -Specify the specialities of HCWs (e.g., nurses, doctors, dentists) as you have only mentioned HCWs in general for some studies. | -We have revised the author names based on your comments.  - We have specified the occupational settings of some of the HCWs in Table 2. However, in some of the included studies, researchers did not provide specific information about the HCWs’ job settings. For this reason, we were unable to report them in the table. | -10-20  -10, 14, 16, 18, 19 |
| ***Table 5: Measures Used***  -Standardize the use of acronyms (e.g., GAD) and provide full terms and detailed explanation in the Result section as suggested in my following comment.  -Avoid redundancy maybe by removing the “main findings” column from Table 2 or justify its presence to prevent confusion and duplication with Table 5. | -Thank you for your comment. We have used both the acronyms and the full names of the measurements in the table for internal consistency.  - We have revised the sentence and clarified the difference between Table 5 from Table 2. | -25, 28, 31, 32  -23 |
| ***Results and Discussion***:  ***Paragraph on Measures Used:***  - Include a detailed paragraph in the results section summarizing the various measures and diagnostic tools used across the included studies. | Thank you for pointing this out. We have included detailed paragraphs that explain the measurements used in the included studies. | -30-31 |
| ***Strengths and Limitations of Included Papers:***  -Clarify the point about the literature gap regarding research on partners and spouses in same-sex relationships, parents, and siblings of HCWs to ensure the readers understand the context clearly. | Thank you for your feedback. We have extended the explanation of the gap in the literature including other family members, close friends, and partners from same-sex relationships of HCWs. | -48 |
| ***Comments from Reviewer #2*** | | |
| For the title I recommend "Impact of Occupational Stress on Healthcare Workers' Families Before and During COVID-19: A systematic review". | Thank you for your recommendation. We have revised our title based on your comment. | -1 |
| In the first paragraph of the introduction, you mentioned statistics for healthcare workers (HCWs) in the UK in 2023, while you motioned statistics globally for 2020? | Thank you for pointing this out. The most up-to-date global information was from 2020, but there was more up-to-date information for the UK as we have cited that as well. Additionally, this study was conducted by a research group in the UK. For these reasons, we preferred to present both statistics. | - |

Thank you again for considering our paper. We hope these amendments address the points raised and very much look forward to hearing further from you.

With kind regards

**Sahra Tekin, Helen Nicholls, Dannielle Lamb, Naomi Glover, and Jo Billings**

**Division of Psychiatry, UCL.**
