# Supplementary material for: Impact of occupational stress on healthcare workers’ family members before and during COVID-19: A systematic review
Source: PLoS One. 2024 Sep 19;19(9):e0308089. doi: 10.1371/journal.pone.0308089 (PMC11412678; doi:10.1371/journal.pone.0308089)
Supplement: Attachment — Submitted filename: .Response to Reviewers.docx [file pone.0308089.s004.docx]

Sahra Tekin

UCL Division of Psychiatry

6^th^ Floor, Maple House

149 Tottenham Court Road

London W1T 7NF

**PLOS ONE**

09 July 2024

Dear Academic Editor,

**LONDON’S GLOBAL UNIVERSITY**

**Re: Manuscript No. PONE-D-24-09879**

**Impact of Occupational Stress on Healthcare Workers' Family Members Before and During the COVID-19: A systematic review**

Thank you to you and your reviewers for your feedback on our manuscript. We are very pleased to submit a revised manuscript incorporating the reviewers’ suggestions. We have provided a point-by-point response to how we have addressed each comment in the table below.

| **Reviewers’ comments** | **Authors’ Response** | **Manuscript page number** |
| --- | --- | --- |
| ***Comments from Reviewer*** | | |
| 1.Please note that your Data Availability Statement is currently missing the repository name and/or the DOI/accession number of each dataset OR a direct link to access each database. If your manuscript is accepted for publication, you will be asked to provide these details on a very short timeline. We therefore suggest that you provide this information now, though we will not hold up the peer review process if you are unable. | Thank you for pointing this out. We have included DOI numbers for each study in the Reference. | -52, 53, and 54 |

Thank you again for considering our paper. We hope these amendments address the points raised and very much look forward to hearing further from you.

With kind regards

**Sahra Tekin, Helen Nicholls, Dannielle Lamb, Naomi Glover, and Jo Billings**

**Division of Psychiatry, UCL.**
